# High-Sensitivity Real-Time Imaging of Dual Protein-Protein Interactions in Living Subjects Using Multicolor Luciferases

**DOI:** 10.1371/journal.pone.0005868

**Published:** 2009-06-12

**Authors:** Naoki Hida, Muhammad Awais, Masaki Takeuchi, Naoto Ueno, Mayuri Tashiro, Chiyo Takagi, Tanuja Singh, Makoto Hayashi, Yoshihiro Ohmiya, Takeaki Ozawa

**Affiliations:** 1 Department of Chemistry, Graduate School of Science, The University of Tokyo, Bunkyo-Ku, Tokyo, Japan; 2 Centre for Cell Imaging, School of Biological Science, The University of Liverpool, Liverpool, United Kingdom; 3 National Institute for Basic Biology, National Institutes of Natural Sciences, Okazaki, Aichi, Japan; 4 Research Institute for Cell Engineering, National Institute of Advanced Industrial Science and Technology (AIST), Osaka, Japan; 5 Graduate School of Medicine, Hokkaido University, Sapporo, Hokkaido, Japan; 6 PRESTO, Japan Science and Technology Agency, Tokyo, Japan; Western Illinois University, United States of America

## Abstract

Networks of protein-protein interactions play key roles in numerous important biological processes in living subjects. An effective methodology to assess protein-protein interactions in living cells of interest is protein-fragment complement assay (PCA). Particularly the assays using fluorescent proteins are powerful techniques, but they do not directly track interactions because of its irreversibility or the time for chromophore formation. By contrast, PCAs using bioluminescent proteins can overcome these drawbacks. We herein describe an imaging method for real-time analysis of protein-protein interactions using multicolor luciferases with different spectral characteristics. The sensitivity and signal-to-background ratio were improved considerably by developing a carboxy-terminal fragment engineered from a click beetle luciferase. We demonstrate its utility in spatiotemporal characterization of Smad1–Smad4 and Smad2–Smad4 interactions in early developing stages of a single living *Xenopus laevis* embryo. We also describe the value of this method by application of specific protein-protein interactions in cell cultures and living mice. This technique supports quantitative analyses and imaging of versatile protein-protein interactions with a selective luminescence wavelength in opaque or strongly auto-fluorescent living subjects.

## Introduction

Although systematic analysis of interacting proteins is performed extensively using the yeast two-hybrid method [1], spatial and temporal information of each protein-protein interaction is crucial for understanding living cells. Protein-fragment complementation assay (PCA) [2], also named bimolecular fluorescence complementation (BiFC) [3]–[7], is useful to visualize subcellular sites of protein-protein interaction under conditions that closely reflect the normal cellular environment. The BiFC analysis generally involves the fusion of split fluorescence protein fragments to a pair of proteins of interest such that neither fragment independently retains fluorescence to a great degree. When proteins of interest mutually interact, two fragments of the fluorescent protein refold correctly and the activity is resumed. BiFC is used for dual interaction of proteins using different spectral characteristics and it also enables for quantitative analysis of dual protein interactions at a single cell level [5]–[7]. Although BiFC analysis is widely used, the chromophore formation of fluorescent proteins and the irreversible reaction of the fragments' complementation limit temporal analysis of protein-protein interactions in living cells [8].

Bioluminescent proteins, luciferases, are used extensively as reporters of many biological functions. It is highly advantageous for the luciferase to emit its photons in the red to near-infrared wavelength, at which tissue attenuation of emitted photons is minimized. Moreover, luciferase reporters are actually more sensitive than fluorescence reporters because they obviate the need for exogenous illumination. External light often bleaches the fluorescence to some extent, yields a higher background fluorescence, perturbs physiology in light-sensitive tissues, and causes phototoxic damage to analyzed cells [9]. Because a bioluminescent reporter protein overcomes those disadvantages, luciferases with distinct characteristics are now used––*Renilla*, firefly, and red and green click beetle luciferases––all of which provide parallel analyses of gene functions in cultured cells and living subjects [10]. We and other groups have demonstrated that PCAs using different luciferases enable this simple assay system to evaluate protein-protein interactions in living cells and mice [11]–[15].

Here, we show an imaging method for spatiotemporal analysis of different protein-protein interactions with significant improvement of both sensitivity and a signal-to-background ratio. Unlike fluorescent proteins, the amino acid sequences of individual luciferases differ greatly among species (Table S1), which hampers cross complementation of luciferase fragments originated from different species. We developed a novel luciferase fragment by random mutagenesis and realized cross complementation between inter- and intra-luciferase fragments with high efficiency. We show potential applications of the luciferase fragments for real-time and dual imaging of kinase-induced interactions of Smad1–Smad4 and Smad2–Smad4 in different stages of a single live *Xenopus laevis* embryo. The obtained results are compared with the previous data; BiFC analysis revealed a subcellular distribution of Smad2–Smad4 at single cell levels during early stages of *Xenopus* embryos [16]. We also present the applicability for visualizing a chemically induced interaction of FKBP-FRB, kinase-induced interactions of IRS-1–p85β, Bad–14-3-3, and Bad–Bcl-2 in cultured cells and living mice.

## Results and Discussion

**Figure 1:**
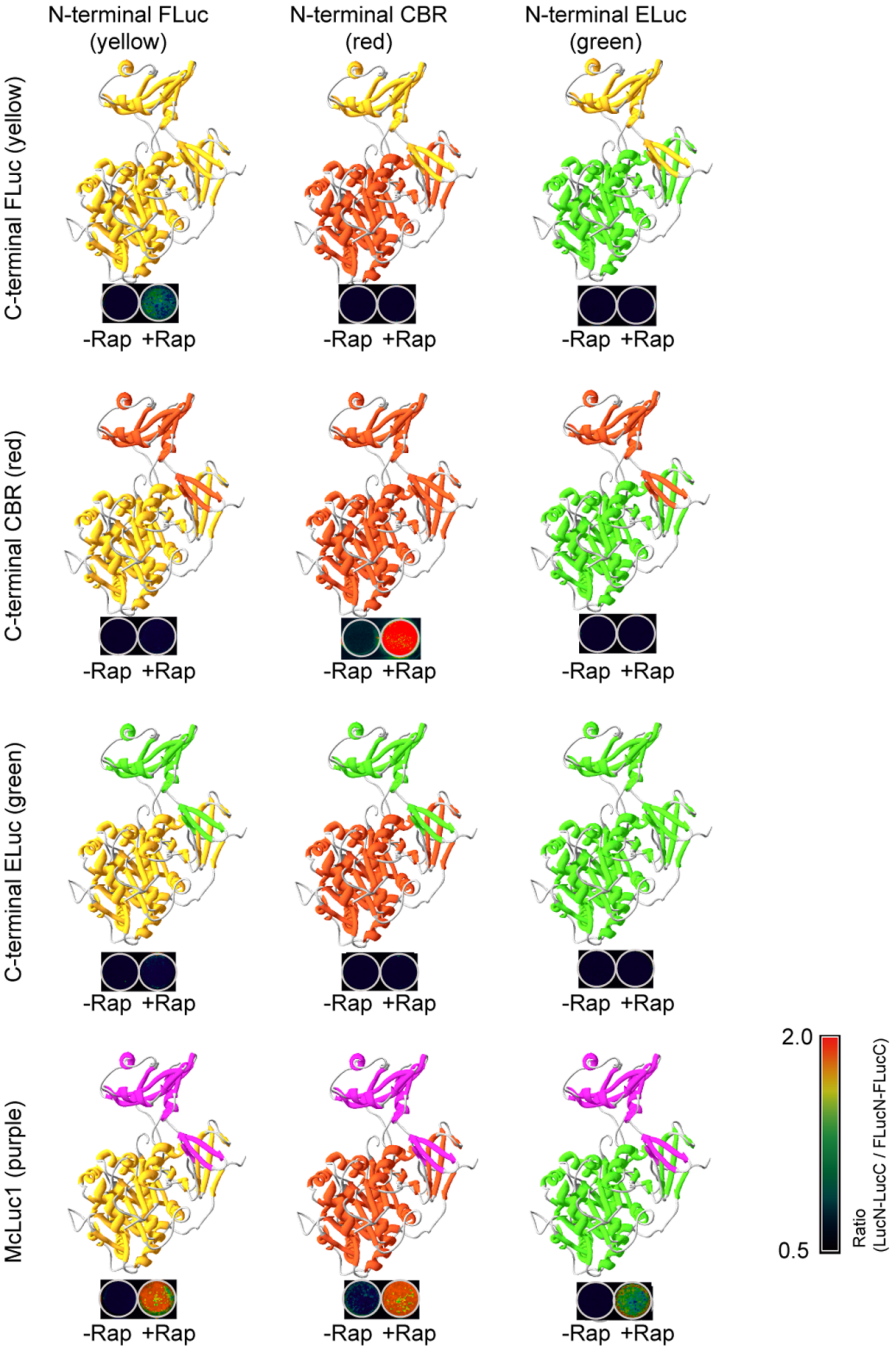
Schematic illustration showing structures of luciferases composed of different luciferase fragments' complementation. Structural models of each luciferase (upper part of each image) are based on the X-ray crystal structure of full-length *Photinus pyralis* (Firefly) luciferase. The N-terminal and C-terminal domains of respective luciferases are shown in different colors. Bioluminescence images of COS-7 cells transfected with plasmids expressing the protein fragments fused to FKBP and FRB are shown below the luciferase structures. The cells were cultured on the 24-well plate and incubated in the presence (+Rap) and absence (−Rap) of rapamycin.

The structure of luciferase from *Photinus pyralis* (FLuc) consists of a large N-terminal domain and a small C-terminal one, which are connected using a flexible linker loop [17] (Figure 1). The substrate D-luciferin is bound in a hydrophobic pocket of the N-terminal domain, although the entrance of the pocket is blocked by the adenosine moiety. The spectral characteristics of luciferase are determined by subtle structural differences of only an amino acid residue in the hydrophobic pocket, whereas the C-terminal domain is used for accelerating the enzymatic reaction [18]. Based on such information, we hypothesized that a common C-terminal fragment of luciferase complements each N-terminal fragment of different-color luciferases when they are brought sufficiently close together.

To examine this, we investigated complementation of split luciferases from firefly (*Photinus pyralis*; Firefly Luc; FLuc), click beetle in green (Brazilian *Cratomorphus distinctus*; Emerald Luc; ELuc) [19], and click beetle in red (Caribbean *Pyrophorus plagiophthalamus*; CBR). Although N-terminal and C-terminal fragments of FLuc have been identified (FLuc(1–413) and FLuc(414–550)) [20], no information exists related to the dissecting sites of ELuc or CBR. We therefore investigated the dissecting sites systematically at amino acid residues from 390 to 410 based on a comparison of the amino acid sequence of FLuc (Table S1). The N-terminal and C-terminal fragments were fused to FK506-binding protein (FKBP) and FKBP-binding domain (FRB) (Figure S1). A pair of the fusion proteins was co-expressed in mammalian cells and the luminescence intensities were examined using a luminometer. Amino-fragments and carboxy-fragments of individual luciferase (ELuc(1–413) and ELuc(394–542), and CBR(1–414) and CBR(395–542) were found to complement sufficiently to recover bioluminescence upon interaction between FKBP and FRB in the presence of rapamycin (Figure 1 and Figure S2). However, no complementation was detected when a combination of different luciferase fragments was used. The spectrum of the complement ELuc was separated from that of CBR (Figure S2 and S3), indicating that complementation of each ELuc and CBR fragments offers the ability to monitor two pairs of protein-protein interactions simultaneously in a single cell.

**Figure 2:**
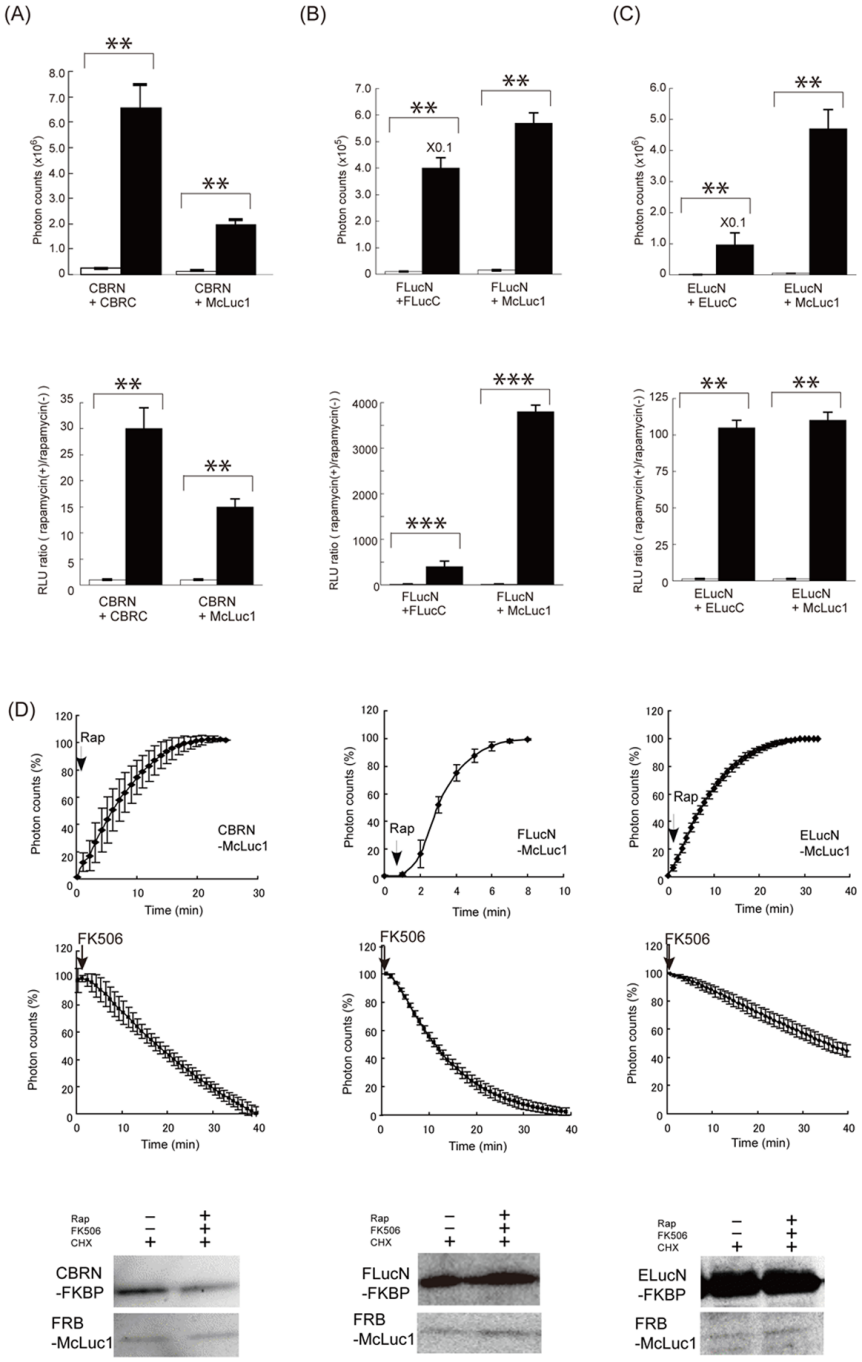
Quantitative and time-course evaluation of rapamycin-induced luciferase complementation. (A–C; upper) Absolute photon counts for COS-7 cells cotransfected with the plasmids of a pair of luciferase fragments fused to FKBP and FRB in the absence (white bars) and presence (black bars) of rapamycin. (A–C; lower) Normalized photon counts for COS-7 cells transiently cotransfected with plasmids expressing the luciferase fragments fused to FKBP and FRB, and *Renilla* luciferase. The *Renilla* luciferase was used to normalize the transfection efficiency. Results are expressed as the relative luminescence unit (RLU) ratio; values of which the luminescence intensity were normalized to the intensity of *Renilla* Luciferase for rapamycin-treated cells were divided by those for rapamycin-untreated cells (black bars). Differences of heights between white and black bars indicate rapamycin-induced luminescence. (D) Reversibility of the complementation between McLuc1 and N-terminal FLuc. The lysates extracted from COS-7 cells expressing FKBP and FRB fused to the luciferase fragments were treated with rapamycin for 1–10 min (50 nM, upper data). The cells were treated successively with different concentrations of FK506 (1 µM, 10 µM, 100 µM) for 0–25 min in the presence of 50 nM rapamycin (middle data). Photon counts were taken every 60 s. The luminescence values were normalized against the maximum luminescence values. Western blotting analysis of the cells including LucN and McLuc1 in the presence or absence of rapamycin and FK506 with cycloheximide (lower). Error bars represent s.d. calculated for three independent samples. (**P<0.01, ***P<0.001)

In complicated protein interaction networks, a protein of interest might interact with proteins of many kinds in single cells. In such cases, it would be more useful if a common C-terminal fragment, which has the ability to complement multiple N-terminal luciferases, were available for the analysis. The fragment of CBR was randomly mutagenized by site-directed mutagenesis [21] to develop such a C-terminal fragment. The products were connected directly with a cDNA of FRB; the fusion proteins were expressed in COS-7 cells including FKBP-connected N-terminal fragments of FLuc, ELuc, or CBR. Among the mutant proteins that were screened, a mutant of the C-terminal fragment including three point mutations, F420I, G421A, E453S, named multiple-complement luciferase fragment (McLuc1), demonstrated the most remarkable properties, which enabled complementation to all N-terminal fragments of FLuc, CBR, and ELuc (Figure 1). Bioluminescence activity of a pair of N-terminal CBR and McLuc1 was recovered 13-fold in the presence of rapamycin in comparison to the background luminescence (Figure 2A). Moreover, McLuc1 was complementary to the N-terminal fragments of FLuc and ELuc, whose respective signals increased 3800-fold and 100-fold upon addition of rapamycin (Figure 2B and C). Remarkably, absolute photon counts of a complement of McLuc1 and N-terminal FLuc exhibited a 12-fold increase compared to those of a native pair of N-terminal and C-terminal FLuc. The signal-to-background ratio upon using McLuc1 was improved to 10-times higher than that of the native one. Similarly, absolute photon counts of McLuc1 with the N-terminal ELuc fragment showed 40-fold stronger than that of N-and C-terminal ELuc fragments (Figure 2C). Also, the bioluminescence intensity of the pair of ELuc fragments was lower than any other native pairs of luciferase fragments (Table S2). Two pairs of luciferase fragments, N-terminal CBR plus C-terminal FLuc and N-terminal ELuc plus C-terminal FLuc, did not emit luminescence in the presence of rapamycin, whereas another pair of different luciferase fragments showed a little extent of bioluminescence. The spectra of N-terminal FLuc, CBR, and ELuc that complement to McLuc1 were almost identical to those of the respective native luciferases (Figure S3), indicating that spectral characteristic of the luciferases are determined by N-terminal domains of the respective luciferases.

We next investigate association and dissociation rates of the new luciferase fragments in vitro. Addition of rapamycin induced rapid increases in the bioluminescence upon complementation of McLuc1 with N-terminal fragments of FLuc, CBR and ELuc. A competitive inhibitor of FK506 prevented the rapamycin-induced bioluminescence generated by all the combinations (Figure 2D). The complex formation and dissociation rates were found apparently similar between all the luciferase fragments. To examine whether the loss of bioluminescence signals in the presence of FK506 is due to complex dissociation or degradation, stability of each luciferase fragment was examined by western blotting analysis (Figure 2D and S4). There was no difference in the abundance of each luciferase fragment over time, indicating that the decreases in the bioluminescence in the presence FK506 were originated from the dissociation of luciferase fragments. All these data demonstrate that McLuc1 is used for a new complementation partner of all N-terminal luciferases tested here and that each pair of luciferase fragments enables multi-color quantitative analysis of protein-protein interactions in living cells.

**Figure 3:**
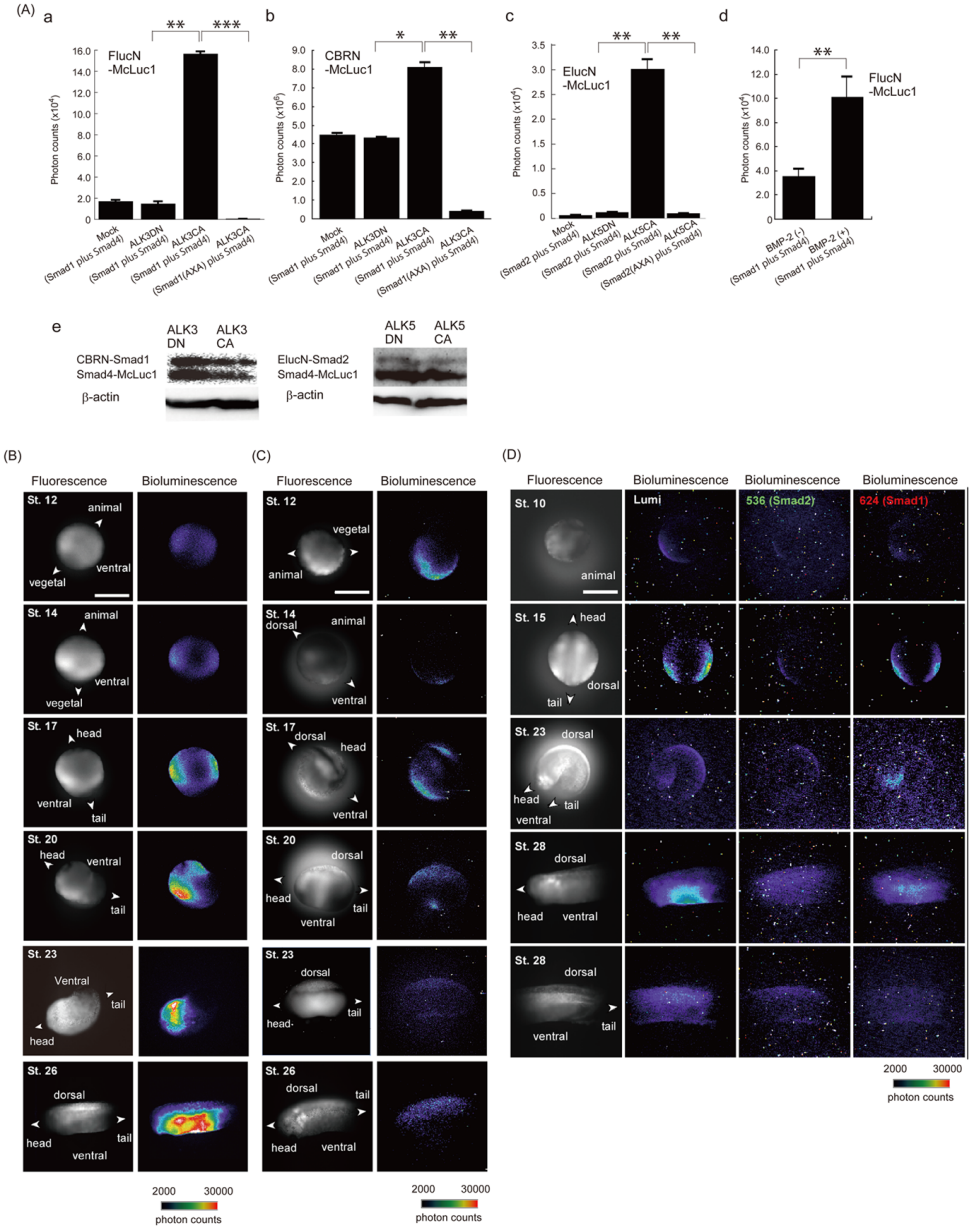
Bioluminescence analysis of Smad1–Smad4 and Smad2–Smad4 interactions. (A) Characterization of the probes. a and b: COS-7 cells were transfected with the plasmids, Smad4-McLuc1 plus FLucN-Smad1 or Smad4-McLuc1 plus CBRN-Smad1, in the absence (Mock) and presence of either ALK3CA or ALK3DN receptor. Smad1(AXA) and Smad2(AXA) indicate double mutants of Smad1(S462A,S464A) and Smad2(S465A,S467A), respectively. The cells were incubated for 12–16 h and the luciferase activities were measured. c: COS-7 cells were transfected with the plasmids, ELucN-Smad2 plus Smad4-McLuc1 or ELucN-Smad2(AXA) plus Smad4-McLuc1 in the absence (Mock) and presence of either ALK5CA or ALK5DN receptor. d: COS-7 cells transfected with the plasmids FLucN-Smad1 and Smad4-McLuc1 were stimulated with BMP-2 (50 nM) for 2 h and luciferase activities were measured. Error bars represent s.d. calculated for three independent samples. e: Western blotting analysis of the expression of Smad1–Smad4 or Smad2–Smad4 protein in the presence of ALK3CA, ALK3DN, ALK5CA or ALK5DN. (*P<0.05, **P<0.01, ***P<0.001) (B)–(D) Real-time bioluminescence images of Smad1–Smad4 and Smad2–Smad4 interactions using CBRN-Smad1 and Smad4-McLuc1 (B), CBRN-Smad2 and Smad4-McLuc1 (C), and CBRN-Smad1, ELucN-Smad2 and Smad4-McLuc1 (D), in a *Xenopus* embryo. RNAs encoding the Smad probes were injected into the animal pole of a 2-cell stage *Xenopus* embryo. The embryo was incubated for 24 h at 13°C and thereafter, digital images of Venus (gray) and bioluminescence (pseudocolor) were acquired using a microscopic system equipped with an EM-CCD camera. Bar, 1 mm.

We applied this technique for a heteromeric complex of Smad1–Smad4 involved in cytoplasmic signaling of the bone morphogenetic protein (BMP) in living cells. We constructed a set of probes consisting of Smad1 connected with the N-terminal fragments of FLuc (FLucN-Smad1) and CBR (CBRN-Smad1) (Figure S1). In addition, a probe of Smad4 connected with McLuc1 (Smad4-McLuc1) was constructed (Figure S1). The probes were expressed in COS-7 cells containing either a constitutively active form of a transmembrane receptor, activin-like kinase 3 (ALK3CA), or its dominant negative form (ALK3DN). When we analyzed bioluminescence intensities of the cells including FLucN-Smad1 and Smad4-McLuc1, the luminescence from the cells expressing ALK3CA was found 8 times higher than that from the cells expressing the ALK3DN or mock-transfected cells (Figure 3A). In the case of expressing CBRN-Smad1 and Smad4-McLuc1, luminescence intensities of the cells showed 2 times higher than that of cells expressing the ALK3DN or mock-transfected cells. When we tested for ELucN-Smad1 and Smad4-McLuc1 under otherwise identical conditions, the luminescence intensity in the presence of ALK3CA increased 12 times higher than that of ALK3DN. The maximum response of the bioluminescence was obtained from the pair of CBRN-Smad1 and Smad4-McLuc1, indicating that the pair of luciferase fragments was useful for sensitively visualizing Smad1–Smad4 interactions in living cells. We next analyzed changes in the abundance of CBRN-Smad1 and Smad4-McLuc1 by western blotting analysis with anti-myc antibody (Figure 3A). No difference was observed in the expression levels of the probes upon expression of ALK3CA or ALK3DN. A Smad1 mutant lacking phosphorylation sites (S462A, S464A) showed negligible luminescence, indicating that phosphorylation of Smad1 induced its interaction with Smad4, thereby resulting in the complement of the luciferase fragments in living cells. These results confirmed that the changes in the bioluminescence intensities were indeed dependent on the interaction of Smad1–Smad4. When COS-7 cells expressing the probes of FLucN-Smad1 with Smad4-McLuc1 were stimulated with BMP-2 for 2 hours, large increases in bioluminescence were observed. This demonstrates that the split luciferase fragments are applicable for detection of inducible protein-protein interactions in living cells.

The Smad1–Smad4 interaction has been shown to play an important role in early developing stages of a *Xenopus laevis* embryo[22]. The embryo has a large amount of fluorescent yolk, which hampers fluorescence imaging because of their spectral overlaps. We applied this bioluminescence technique for a time-lapse imaging of the interaction in a single *Xenopus* embryo. We synthesized mRNAs from cDNA constructs of CBRN-Smad1 and Smad4-McLuc1, and microinjected the mRNAs into two diagonal blastomeres of the 2-cell embryo. The mRNA of a yellow fluorescent protein named Venus was also injected for visualizing the whole shape of the embryo. After the embryo was set on a glass dish and soaked in a solution including D-luciferin, embryonic development was monitored over 24 h using a handmade microscopic system equipped with a cooled charge-coupled device (CCD) camera (Figure 3B and Movie S1). When a sibling embryo reached stage 10, a weak but significant signal of bioluminescence was detected in the lateral sides of ventral region. The signal became intense of stage 14, and was stronger from stage 17 to stage 26. Localization of the bioluminescence signals was the same as that of BMP signals detected with anti-phosphorylated Smad1 antibody [22]. It is noteworthy that specific patterns of bioluminescence signals in the embryo do not represent distribution of the mRNA, but rather real endogenous BMP signals because the same embryo including the Venus mRNA showed almost ubiquitous expression patterns among all cells over time. To confirm this further, we obtained fluorescence images of the fusion proteins, Venus-Smad1 and Venus-Smad4, expressed in *Xenopus* embryo in order to examine their expression levels and localization (Figure S5). The Venus-fusion Smads were expressed ubiquitously in *Xenopus* embryo at stage 10, 17 and 23. We also examined the auto-fluorescence and auto-bioluminescence imaging of un-injected *Xenopus* embryos (Figure S5). The fluorescence intensity of yolk was 3-times lower than that of Venus or Venus-fusion Smads. In contrast, the auto-bioluminescence signals were negligible, demonstrating that luciferase complementation has a property of higher signal-to-background ratio upon using auto-fluorescent samples.

Next, we investigated the Smad2–Smad4 interaction induced by TGF-β. A probe consisting of Smad2 connected with N-terminal ELuc (ELucN-Smad2) was constructed (Figure S1) and expressed with Smad4-McLuc1 in COS-7 cells. The cells expressing ALK5CA revealed strong bioluminescence, whereas the cells expressing ALK5DN were silent (Figure 3A). The Smad2 was replaced by a Smad2 mutant lacking phosphorylation sites (S465A, S467A) and performed the same experiments. The cells showed little bioluminescence even in the presence of ALK5CA. Western blotting analysis revealed no significant difference in the expression level of the probes when ALK5DN or ALK5CA was expressed in the cells, confirming that phosphorylation of Smad2 induced its interaction with Smad4 in COS-7 cells. We further characterized the Smad2–Smad4 interaction induced by TGF-β in a single *Xenopus* embryo. We injected mRNA of CBRN-Smad2 instead of CBRN-Smad1 and performed under otherwise identical conditions. Strong bioluminescence was found in both lateral sides of the ventral region during stages 10–17 (Figure 3C and Movie S2). In addition, a bioluminescence signal appeared in the dorsal region of trunk at stage 17; thereafter the signal intensified along the entire dorsal region from head to tail. The signal was sustained on the dorsal side up to stage 26 and gradually faded away, indicating dissociation between Smad2 and Smad4. Fluorescence images of Venus-Smad2 in *Xenopus* embryos showed almost ubiquitous expression patterns (Figure S5), indicating that the obtained bioluminescence signals were due to the complementation of CBRN with McLuc1 induced by the Smad2–Smad4 interaction in the embryo.

The specific patterns of the observed bioluminescence at stage 12 indicate activation of an endogenous TGF-β signaling from the marginal zone. A previous study using BiFC has demonstrated an accumulation of Smad2–Smad4 interaction in marginal zone cells of vegetal hemisphere in a subcellular level [16]. Although spatial resolution of the present data on the whole embryo was not so high as the data using BiFC because of low photon emission, the spatial pattern of Smad2–Smad4 interaction was almost consistent with the previous data obtained by BiFC analysis. This indicates that luciferase complementation is suitable for temporal analysis of protein-protein interaction, whereas BiFC analysis is better for spatial analysis of the interaction with higher resolution.

To test whether it is possible to detect the interactions of Smad1–Smad4 and Smad2–Smad4 simultaneously in a single *Xenopus* embryo, mRNAs of CBRN-Smad1, ELucN-Smad2 and Smad4-McLuc1 were injected into the embryo. Specific images originated from bioluminescence signals were obtained in the absence and presence of band-pass filters (536±10 nm and 624±25 nm), although the intensities of the bioluminescence were less than 10% (Figure 3D). At the stage 23, anterior region nearby cement gland of embryo showed strong bioluminescence originated from the Smad1–Smad4 interaction. Bioluminescence from the Smad2–Smad4 interaction was only detected at the dorsal side. The specific patterns of the bioluminescence were identical to those obtained from the independent experiments shown in Figure 3B and 3C. The auto-bioluminescence background signals were negligible during early stages of *Xenopus* embryos. These results reflect the usefulness of McLuc1 and N-terminal fragments of the luciferases for dual imaging of different protein-protein interactions with a third shared protein in the developing stages of a single embryo.

**Figure 4:**
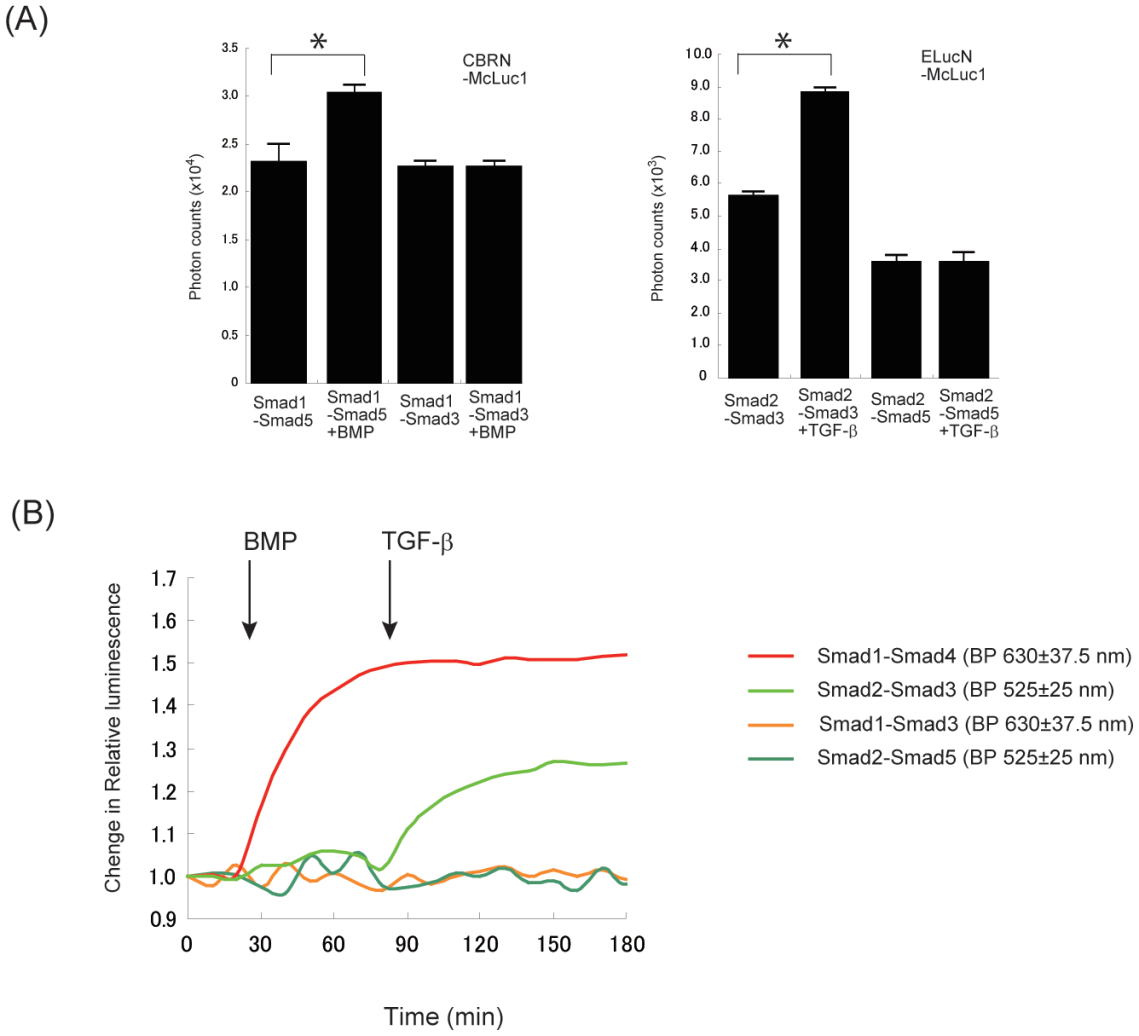
Dual-color assay for Smad1–Smad5 and Smad2–Smad3 interactions using different fragments of luciferases. (A) Ligand-induced Smad1–Smad5 and Smad2–Smad3 interactions. COS-7 cells were cotransfected with CBRN-Smad1, Smad5-McLuc1, ELucN-Smad2 and Smad3-McLuc1 and their bioluminescence intensities were obtained with a luminometer. (*P<0.05) (B) Analysis of bioluminescence with different combination of Smads. COS-7 cells were cotransfected with either CBRN-Smad1 and Smad3-McLuc1 or ELucN-Smad2 and Smad5-McLuc1. Representative data were shown.

Detection of two discrete pairs of interactions in living cells is also important for resolving complex protein networks. We applied the multicolor-luciferase fragments for dual imaging of Smad1–Smad5 and Smad2–Smad3 interactions in living cells. It has been shown that Smad5 and Smad3 are phosphorylated by ligand-induced ALK3 and ALK5, respectively [23]. When Smad2 is phosphorylated by TGF-β-induced activin-like kinase, Smad2 forms heterotrimer with Smad3 and Smad4 [24]. Smad1 is also considered to interact with Smad5 as well as the case of Smad2–Smad3 interaction. Each Smad3 and Smad5 was connected with McLuc1 and two pairs of interactions, Smad1–Smad5 and Smad2–Smad3, were visualized in COS-7 cells using CBRN-Smad1 and ELucN-Smad2. Bioluminescence signals from BMP-induced Smad1–Smad5 and TGF-β-induced Smad2–Smad3 interactions were obtained with green and red band-pass filters (Figure 4). Upon stimulation with 50 nM BMP-2, red bioluminescence intensity was found to increase over time. When 20 nM TGF-β was added to the cells 50 min after BMP stimulation, only green bioluminescence increased whereas red bioluminescence was silent. A different pair of CBRN-Smad1 and Smad3-McLuc1 or ELucN-Smad2 and Smad5-McLuc1 did not show any bioluminescence signals. These results demonstrate that the combination of multicolor N-terminal fragments of click beetle luciferases with McLuc1 makes it possible to monitor discrete pairs of protein-protein interactions in the same living cells.

**Figure 5:**
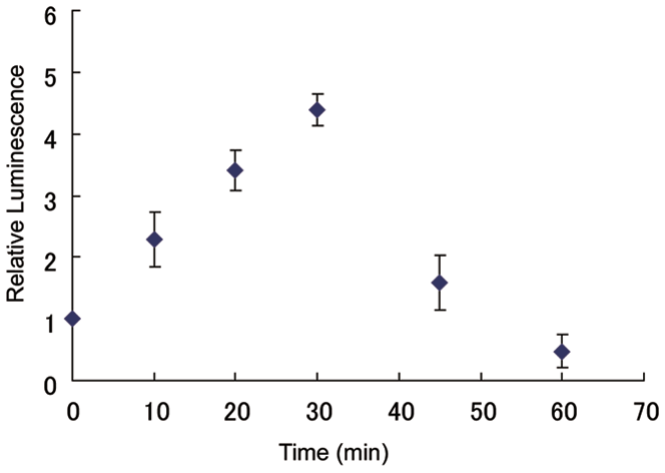
Analysis of IRS1-p85β interaction based on luminescence intensities of McLuc1 and FLucN complementation. The CHO-IR cells were transiently transfected with IRS1-McLuc1 and FLucN-p85β; luciferase activities were measured at each time point. Error bars represent s.d. calculated for three independent samples.

To show applicability for another interaction with reversible and transient character, we used the McLuc1 and N-terminal fragment of FLuc to assay an interaction of IRS-1 with PI3-Kinase (Figure S1), which are known to regulate insulin signal cascade [25]. In the presence of insulin, IRS-1 is phosphorylated, thereby interacting with p85β of PI3-Kinese. The IRS-1 and the p85β were fused to McLuc1 and N-terminal fragment of FLuc, respectively. cDNAs of the fusion proteins were cotransfected into CHO-IR cells expressing insulin receptor [26], and the bioluminescence intensities were examined by a luminometer. Upon stimulation with 100 nM insulin, a large increase in bioluminescence signals was observed (Figure 5), indicating that phosphorylated IRS-1 interacted with p85β. Subsequently, the medium including insulin was replaced by a fresh medium without insulin and the cells were incubated for 30 minutes thereafter. The bioluminescence signal showed a gradual decrease due to the dephosphorylation of IRS-1. This result indicates that the McLuc1 is applicable for monitoring relatively rapid and reversible interactions in living cells.

**Figure 6:**
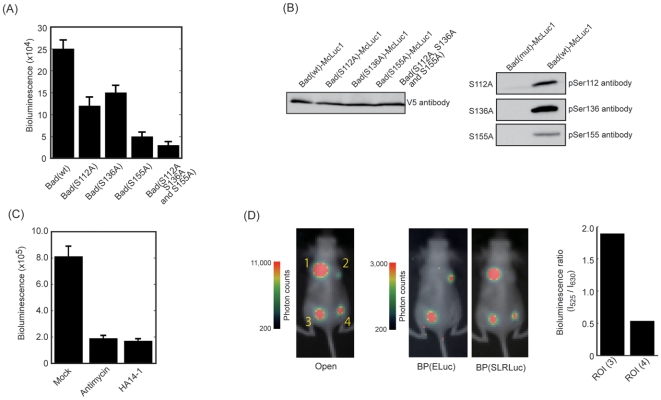
Analysis of Bad phosphorylation based on luminescence intensities of the McLuc1 and N-terminal ELuc complementation. (A) Results of analyses of interactions between 14-3-3 and Bad mutants. The COS-7 cells were transiently transfected with Bad–McLuc1 and ELucN–14-3-3; luciferase activities were tested. Error bars represent s.d. calculated for three independent samples. (B) Results of Western blotting analysis of Bad phosphorylation. Expression levels of Bad and its mutants were determined using immunoblotting with the anti-V5 antibody (left). Mutations of Bad at S112A, S136A, and S155A were confirmed by immunoblotting with the respective antibodies (right). (C) An inhibitory effect of antimycin or HA14-1 on the bioluminescence was developed by the Bad-Bcl-2 interaction. Antimycin (10 µM) or HA14-1 (10 µM) was added to the cells after transfection and incubated for 20 h. The cells were harvested and the photon counts were analyzed. Error bars represent s.d. calculated for three independent samples. (D) Dual color imaging of mice with a single substrate. The images shown are superimposed on the optical CCD bioluminescence image without a filter (Open) or with a band-pass filter of BP(ELuc) (525±25 nm) or BP(SLRLuc) (630±37.5 nm). A nude mouse was imaged after implantation of COS-7 cells that had been transiently transfected with plasmids SLRLuc (site 1), Bad–McLuc1 and ELucN–14-3-3 (site 2), SLRLuc plus Bad–McLuc1 and ELucN–14-3-3 (site 3), and SLRLuc plus Bad(S112A, A136A, A155A) –McLuc1 and ELucN–14-3-3 (site 4). To obtain photon flux information from mice, the bioluminescence intensity was shown as pseudocolors. Photon counts with BP(ELuc) divided by those with BP(SLRLuc) are shown at the right side of the image.

We further used the McLuc1 and N-terminal fragments of luciferases to assay phosphorylation of Bad, which is known to regulate cell survival [27]. In the presence of growth factors, Bad is phosphorylated, thereby interacting with the 14-3-3 protein. Upon deprivation of growth factors, Bad is dephosphorylated. Subsequently, it binds to one of the Bcl-X_L_ family members, Bcl-2. The Bad protein was fused to McLuc1 (Bad–McLuc1), whereas the 14-3-3 and Bcl-2 proteins were fused, respectively, to N-terminal fragments of ELuc (ElucN–14-3-3) and FLuc (FLucN–Bcl-2) (Figure S1). A strong bioluminescence signal was detected from COS-7 cells transfected with ELucN–14-3-3 and Bad–McLuc1 (Figure 6A), indicating that Bad interacts endogenously with 14-3-3. To confirm that the bioluminescence signal was indeed triggered by interaction of Bad and 14-3-3, we constructed Bad mutants in which all the key serine residues; S112, S136, and S155; which play an important role for interaction of Bad with 14-3-3, were replaced with alanine. Upon expression of 14-3-3 and the Bad mutants in the cells, bioluminescence signals were reduced significantly. A triple mutant of Bad (S112A, S136A, S155A) showed a negligible interaction with 14-3-3 (Figure 6A). Phosphorylation of the serine residues (S112, S136, S155) in Bad is necessary for interaction of Bad with 14-3-3. The lack of these phosphorylation sites was confirmed using Western blotting with respective antibodies (Figure 6B). The cDNA construct of Bad–McLuc1 was further used for the analysis of interaction with Bcl-2. When the Bad–McLuc1 and FLucN–Bcl-2 were expressed in COS-7 cells, a strong bioluminescence signal was obtained. Addition of Bcl-2 inhibitors, antimycin or HA14-1, caused reduction of the bioluminescence signals (Figure 6C), demonstrating that three fragments of McLuc1, N-terminal ELuc and FLuc are useful for detecting interactions of a protein associated with multiple distinct protein partners.

We also examined the usefulness of the probe for visualizing Bad phosphorylations in living mice. For bioluminescence imaging *in vivo* with living mice, *Renilla* luciferase and its substrate coelenterazine are used to normalize the transfection efficiency and the number of implanted cells. However, that mode of analysis is limited by qualitative evaluation because of the differences of temporal fluctuation and inhomogeneous distribution between D-luciferin and coelenterazine. This limitation can be overcome if the same D-luciferin is used instead of coelenterazine for the dual analysis. We selected a luciferase derived from *Phrixothrix* railroad worm (SLRLuc) as an internal control [28] to show the proof-of-principle that McLuc1 is applicable for two-color monitoring with a single substrate, D-luciferin. The emission maximum of SLRLuc is 630 nm, of which the spectrum is separated completely from that of complementary fragments of McLuc1 and N-terminal ELuc (Figure S3). To show dual color imaging with a single substrate, we implanted COS-7 cells that had been cotransfected with three plasmids on the backs of mice: Bad connected with McLuc1, 14-3-3 connected with N-terminal ELuc, and SLRLuc. Then D-luciferin was injected intraperitoneally in the mice and bioluminescence images were taken using a cooled CCD camera. The image contained the sum of the light from two luciferases, SLRLuc, and complementary fragments of N-terminal ELuc and McLuc1 (Figure 6D). We next passed the emitted photons through a green filter (BP525±25 nm), thereby detecting photons only from ELuc. In contrast, when the filter was replaced into a red filter (BP 630±37.5 nm), the image was separated from the image that was taken with the green filter, confirming that SLRLuc is suitable as an internal control for dual-color bioluminescence imaging with the single D-luciferin.

In conclusion, we developed a novel luciferase fragment of McLuc1. It has the unique ability to complement multiple N-terminal luciferases with different spectral characteristics. Sensitivities of McLuc1 which complements to N-terminal fragments of FLuc and ELuc were improved 12-fold and 40-fold higher than those of native pairs of luciferase fragments. In addition, the complementation reaction with McLuc1 occurs within a few minutes and is reversible. Because of its high sensitivity and reversibility, the luciferase fragments enable spatiotemporal and simultaneous imaging of protein-protein interactions of two kinds in opaque and auto-fluorescent living subjects. Thus, the present method using luciferase-fragment complementation provides a sensitive and quantitative method to assess two discrete pairs of protein interactions or three protein interactions with a third shared protein. Such dual assay system for multiple protein-protein interactions might be constructed if we would use mutant firefly luciferases with different spectral properties. Although the approach with firefly luciferases seems to be straightforward, a drawback exists: A spectrum of firefly luciferase is known to change in a pH-dependent manner, which may hamper spectral deconvolution or precise photon counts with specific filters. By contrast, crick beetle luciferases and their mutants have a specific character of pH independence on the spectra. Therefore, the use of McLuc1 with different N-terminal luciferase fragments allows precise and quantitative detection of multiple protein interactions in living cells. The imaging technique using luciferase fragments might greatly facilitate imaging analysis of protein networks *in vitro* and *in vivo* in a wider range of organisms.

## Materials and Methods

### Construction of mammalian expression vectors

All cDNAs were cloned with pBlueScript (Stratagene) using standard methods, with sources of FLuc and CBR (Promega Corp.), and ELuc (Toyobo Co. Ltd., Japan). The cDNAs encoding FLuc (1–413), FLuc (414–550), ELuc (1–413), ELuc (394–542), CBR (1–414), CBR (395–542), FKBP, FRB, 14-3-3 (1–244), Bcl-2, Bad (1–204), Smad1, Smad2, Smad3, Smad4, Smad5, IRS-1 and p85β were generated by polymerase chain reaction (PCR) to attach a Kozak sequence and restriction enzyme sites. Mutants of Bad(S112A), Bad(S136A), Bad(S155A), Bad(S112A,S136A,S155A), and Smad1(S462A,S464A) and Smad2 (S465A, S467A) were generated using a mutagenic PCR technique. All PCR fragments were sequenced using a genetic analyzer (ABI310; Applied Biosystems). The cDNA fragments used for cultured COS-7 cells were subcloned into a mammalian expression vector, pcDNA4/V5-His(B) (Invitrogen Corp., Carlsbad, CA). The constitutive active form and dominant negative form of activin-like kinase 3 (ALK3) and activin-like kinase 5 (ALK5) (gifted from Dr. T Imamura, The Cancer Institute of JFCR, Japan) were subcloned into a mammalian expression vector, pcDNA3 (Invitrogen Corp.).

### Cell culture and Transfection

For this study, COS-7 cells were sub-cultured in 12-well plates in Dulbecco's modified medium supplemented with 10% fetal bovine serum (FBS) and 1% penicillin-streptomycin (P/S) at 37°C in a 5% CO_2_ incubator. Cells in 24-well plates were transfected with an expression vector including the probe constructs in the presence of Lipofectamine 2000 (Gibco BRL).

### Western blotting

The COS-7 cells were plated on 6-well plates and transiently transfected with each combination of LucN-FKBP and FRB-LucC or Bad(wt)–McLuc1, Bad(S112A)–McLuc1, Bad(S136A)–McLuc1, Bad(S155A)–McLuc1, or FLucN–14-3-3. At 24 h transfection, the cells expressing FKBP-FRB probes were incubated with 20 µM cycloheximide. Three hours later, the cells were lysed with a buffer with D-Luciferin (Steady-Glo system, Promega). This sample is Rap(−). After lysis of the cells, 50 nM rapamycin was added. When the samples showed a maximal luminescence response to rapamycin, 100 nM FK506 was added. The sample decreasing bioluminescence were mixed with sample buffer (125 mM Tris pH 6.8, 10% glycerol, 4% SDS, 0.006% Bromophenol blue, 1.8% beta-mercaptoethanol). This sample is Rap+, FK506+, CHX+. At 16–24 h after transfection, the cells expressing Bad–14-3-3 probes were lysed with a sample buffer (125 mM Tris pH 6.8, 10% glycerol, 4% SDS, 0.006% Bromophenol blue, 1.8% beta-mercaptoethanol). Then samples of the lysates were subjected to Western blotting using anti-V5 (Sigma) and anti-Bad (pBad(S112), pBad(S136) and pBad(S155); Cell Signaling Technologies) antibodies [1∶5000 in 1% skimmed milk in TBST (50 mM Tris-HCl pH 8.0, 150 mM NaCl, 0.05% Tween 20)] and alkaline-phosphatase-labeled anti-mouse antibody (GE Healthcare) (1∶4000 in 1% skimmed milk in TBST). The phosphatase activities were visualized using an ECL advance Western blotting detection kit (GE Healthcare) with an image analyzer (LAS-1000 plus Fuji Photo Film Co.).

### Luminescence assay

The COS-7 cells were transfected with the plasmids including N-terminal and C-terminal fragments of luciferase and incubated for 12–16 h. The cells were stimulated with rapamycin (final concentration, 100 nM), and incubated for 4 h. As its negative control, the COS-7 cells were not stimulated by rapamycin, but were subjected to all other experimental procedures. The luciferase activities were measured according to the manufacturer's protocol (Promega Corp.). The time for measuring each luciferase activity was 30 s. All measurements were performed using a luminometer (MiniLumat LB9506; Berthold GmbH & Co. KG) and were made in triplicate with different wells of culture plates. Results are presented as average ratios with standard deviations.

### Imaging

We set up a dual system for fluorescence and bioluminescence imaging on an upright microscope (BX61; Olympus Corp.) with 10× dipping objective (0.40 NA) for *Xenopus* embryo and 20× dipping objective (0.10 NA) for plant cells. We selected a 60 W metal-halide light source for illumination of fluorescent proteins, and filter sets for Venus (excitation 490±10 nm; emission 530±10 nm). For bioluminescence imaging, we used only band-pass emission filters (536±10 nm and 624±25 nm). Emitted light from the sample was passed through a lens attachment (U-TV0.25XC; Olympus Corp.) in front of a camera. Digital images were acquired using a cooled (set at −80°C) EM-CCD camera (ImagEM; Hamamatsu Photonics K.K.). The filters and camera control were adjusted automatically using software (Meta Morph; Universal Imaging Corp.). Stray light was cut off by turning off the electric system and covering it tightly with foil. The imaging system was used in a dark room (A-3542-03; Hamamatsu Photonics K.K.).

### Analysis and imaging of Smad1–Smad4 and Smad2–Smad4 interactions in living cells and a *Xenopus* embryo

The COS-7 cells were transfected with plasmids, Smad4-McLuc1 plus LucN-Smad1 (LucN: FLucN, CBRN or ELucN) or Smad4-McLuc1 plus ELucN-Smad2 in the absence (Mock) and presence of either ALK3CA, ALK3DN, ALK5CA or ALK5DN. A Smad1 mutant of Smad1(AXA) and a smad2 mutant of Smad2(AXA) were constructed. The mutants were connected with LucN instead of Smad1 or Smad2, and expressed in the COS-7 cells. The cells were incubated for 12–16 h; the luciferase activities were measured for 30 s with a luminometer (MiniLumat LB9506; Berthold GmbH & Co. KG). Data acquisitions were made in triplicate with different wells of culture plates. Results are presented as averages with standard deviations for three independent experiments.

To visualize the interaction in a *Xenopus* embryo, CBRN-Smad1, ELucN-Smad2, CBRN-Smad2, and Smad4-McLuc1 were subcloned into the pCS2 vector. Using the vector, capped mRNAs were synthesized *in vitro* using a kit (mMACHINE Sp6; Ambion Inc.) according to the manufacturer's protocol. The RNAs were injected into the animal pole of the 2-cell stage *Xenopus* embryos in a 3% Ficoll, 0.1% Steinberg's solution. The embryos were incubated for 24 h at 13°C. After incubation, a single embryo was dipped in a 0.1× MMR buffer (0.1 M NaCl, 2.0 mM KCl, 1 mM MgSO_4_, 2 mM CaCl_2_, 0.1 mM EDTA, 5 mM Hepes, pH 7.8) including 1 mM D-luciferin, which was set on a stage of the microscopic system. To acquire bioluminescence images, the exposure time was set to 30 s (Smad1–Smad4 interaction) and 5 min (Smad2–Smad4 interaction) for single wavelengths, and at 60 min (Smad1–Smad4 interaction) and 90 min (Smad2–Smad4 interaction) for dual imaging.

### Detection of IRS-1 and PI3-Kinase interactions

To detect an interaction of IRS-1 with PI3-Kinase, we used a pair of luciferase fragments, McLuc1 and N-terminal fragment of FLuc. The CHO-IR cells expressing insulin receptor were cultured in 12 well plates. The cells were transfected with cDNAs of IRS-1–McLuc1 and FLucN–p85β and pTK-RLuc (Promega Corp.). After incubation for 24 h, the cells were stimulated with 100 nM insulin. The luminescence signals were measured in each time point. All measurements were performed using a luminometer (MiniLumat LB9506). The obtained luminescence from D-luciferin (LF) normalized against the luminescence from coelenterazine (LR) was termed as the relative light unit (RLU; RLU = LF/LR). Data were made in triplicate with different wells of culture plates. Results were presented as average ratios with standard deviations.

### Imaging of Bad–14-3-3 and Bad–Bcl-2 interactions

For imaging the interaction between Bad and 14-3-3 in living mice, we used N-terminal ELuc and McLuc1 as complementation partners. Furthermore, SLRLuc (Toyobo Co. Ltd.) was used as an internal control. The COS-7 cells were cultured in 10 cm dishes. Each dish was transfected with Bad–McLuc1 and ELucN–14-3-3 plus SLRLuc, Bad(S3A)–McLuc1, ELucN–14-3-3 and SLRLuc. The cells were harvested after incubation for 18–24 h. The cells were suspended in phosphate buffer saline. Then an aliquot of 1×10^6^ cells was implanted in anesthetized BALB/c nude mice (females, 4 weeks old, ca. 15 g body weight). Fifteen minutes after cell implantation, D-luciferin, 3 mg dissolved in 100 µL of PBS, was injected i.p. Ten minutes after injection of D-luciferin, bioluminescence images were taken using a cooled CCD camera (Versarray 1300B, Princeton Instruments Inc.) with or without a filter (BP(ELuc); 520±25 nm, BP(SLRLuc); 630±37.5 nm, Chroma Technology Corp.). Image processing was performed using imaging software (SlideBook 4.1; Intelligent Imaging Innovation Inc.). To quantify the measured luminescence, regions of interest were drawn over the cell-implanted areas. Then, luminescence intensities were evaluated.

### Statistical analysis

Where indicated, two-tailed Student's *t-*tests (Calculated in Microsoft Excel) were used. All summary bar graphs are presented as mean±s.d., with significance denoted as follows: *P<0.05, **P<0.01, ***P<0.001.

## Supporting Information

Figure S1 Schematic structures of major constructs. 14-3-3 and Bad indicate the cDNA sequences encoding the 1–244 and 1–204 amino acids of 14-3-3 and Bad proteins, respectively. Enzyme sites and the length of the cDNA constracts of Bad mutants, Bad(S112A), Bad(S136A), Bad(S155A), Bad(S112A, S136A, S155A) are the same as those of Bad(wt). V5, V5 epitope; Myc, Myc epitope.
(3.89 MB TIF)

Figure S2 Bioluminescence imaging of COS-7 cells transfected with plasmids expressing the luciferase fragments fused to FKBP and FRB. The cells were cultured on the 24-well plate in the presence (+) and absence (−) of rapamycin. Each image was taken without a filter (OPEN) or with a band-path filters (BP 525±25 nm and BP630±37.5 nm) by using a cooled CCD camera (Versarray: 1300B, Prinston Instruments) after addition of D-luciferin. Image processing was performed by a SlideBook 4.1 imaging software.
(2.99 MB TIF)

Figure S3 Bioluminescent spectra of cells expressing McLuc1 and N-terminal fragments of FLuc (FLucN), ELuc (ELucN) and CBR (CBRN). Spectra indicate ELucN plus McLuc1 (green, a), FLucN plus McLuc1 (orange, b), CBRN plus McLuc1 (red, c), and SLRLuc (dark red, d).
(1.29 MB TIF)

Figure S4 Reversibility of the complementation between N- and C-terminal fragments of luciferases. An N-terminal fragment of luciferase (LucN; FLucN, CBRN or ELucN) was connected with FKBP, whereas a C-terminal fragment of luciferase (Luc-C; ELucC, CBRC or FLucC) was connected with FRB. Each pair of N- and C-terminal luciferases was expressed in COS-7 cells. The cell lysates were treated with rapamycin for 25 min (50 nM, A–C; upper data). The cells were treated successively with FK506 (100 ÂµM) for 40 min (A–C; middle data). Photon counts were taken every 60 s. The luminescence intensites were normalized against the maximum luminescence values. Error bars represent s.d. calculated for three independent samples. (A–C; lower) Western blotting analysis of the expression of LucN with McLuc1 in the presence or absence of rapamycin and FK506 with cycloheximide.
(1.30 MB TIF)

Figure S5 Fluorescence imaging of single living Xenopus embryos including Venus-fusion Smad1, Smad2 and Smad4 (left). RNA encording the Venus-fusion Smads were injected into the animal pole of a 2-cell stage Xenopus embryo. The injected embryo was incubated for 24 h at 13°C. At the indicated stages, fluorescence images were taken under fluorescence microscope equipped with a CCD camera. As the control experiments, auto-fluorescence and auto-bioluminescence images of Xenopus embryo were taken under the identical conditions.
(4.94 MB TIF)

Table S1 Comparison of the amino acid sequences between FLuc, CBR and ELuc.
(0.20 MB PDF)

Table S2 Maximal bioluminescence intensities of complement luciferase fragments in the presence of rapamycin.
(0.01 MB PDF)

Movie S1 Three mRNAs of CBRN-Smad1, Smad4-McLuc1 and Venus were microinjected into two diagonal blastomeres of the 2-cell embryo. The embryo was set on a glass dish and dipped in a solution including 1 mM D-luciferin. The embryonic development was monitored over 24 hours under the fluorescence and bioluminescence upright microscope. Obtained digital images of bioluminescence (pseudocolor) were overlaid with the images of fluorescence (gray) at each time.
(0.49 MB AVI)

Movie S2 Three mRNAs of CBRN-Smad2, Smad4-McLuc1 and Venus were microinjected into two diagonal blastomeres of the 2-cell embryo. The embryo was set on a glass dish and dipped in a solution including 1 mM D-luciferin. The embryonic development was monitored over 12 hours under the fluorescence and bioluminescence upright microscope. Obtained digital images of bioluminescence (pseudocolor) were overlaid with the images of fluorescence (gray) at each time.
(0.68 MB AVI)
