# Supplementary figures and images for: High-Sensitivity Real-Time Imaging of Dual Protein-Protein Interactions in Living Subjects Using Multicolor Luciferases
Source: PLoS One. 2009 Jun 12;4(6):e5868. doi: 10.1371/journal.pone.0005868 (PMC2697115; doi:10.1371/journal.pone.0005868)

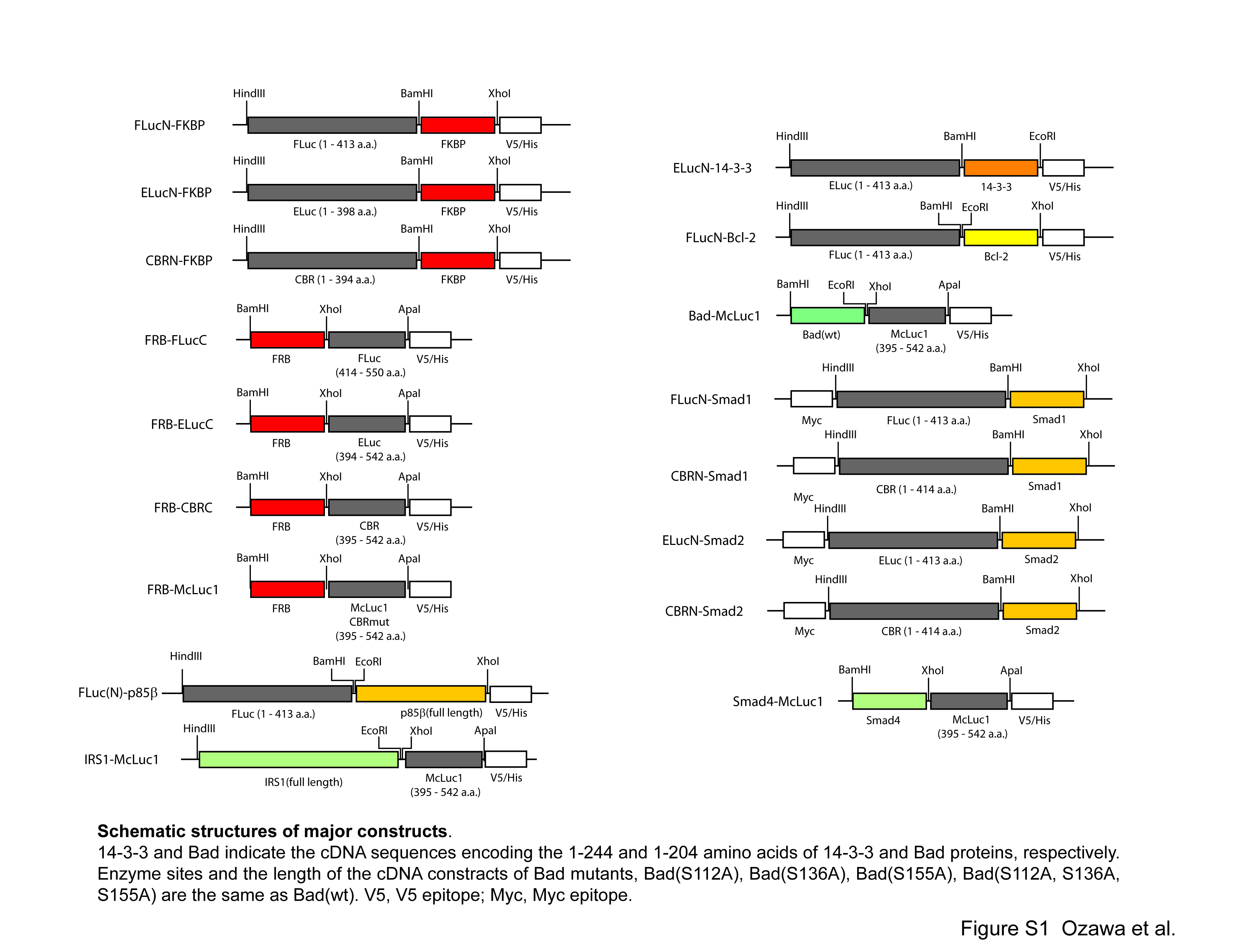

Supplement: Figure S1 — Schematic structures of major constructs. 14-3-3 and Bad indicate the cDNA sequences encoding the 1–244 and 1–204 amino acids of 14-3-3 and Bad proteins, respectively. Enzyme sites and the length of the cDNA constracts of Bad mutants, Bad(S112A), Bad(S136A), Bad(S155A), Bad(S112A, S136A, S155A) are the same as those of Bad(wt). V5, V5 epitope; Myc, Myc epitope. (3.89 MB TIF) [file pone.0005868.s001.tif]

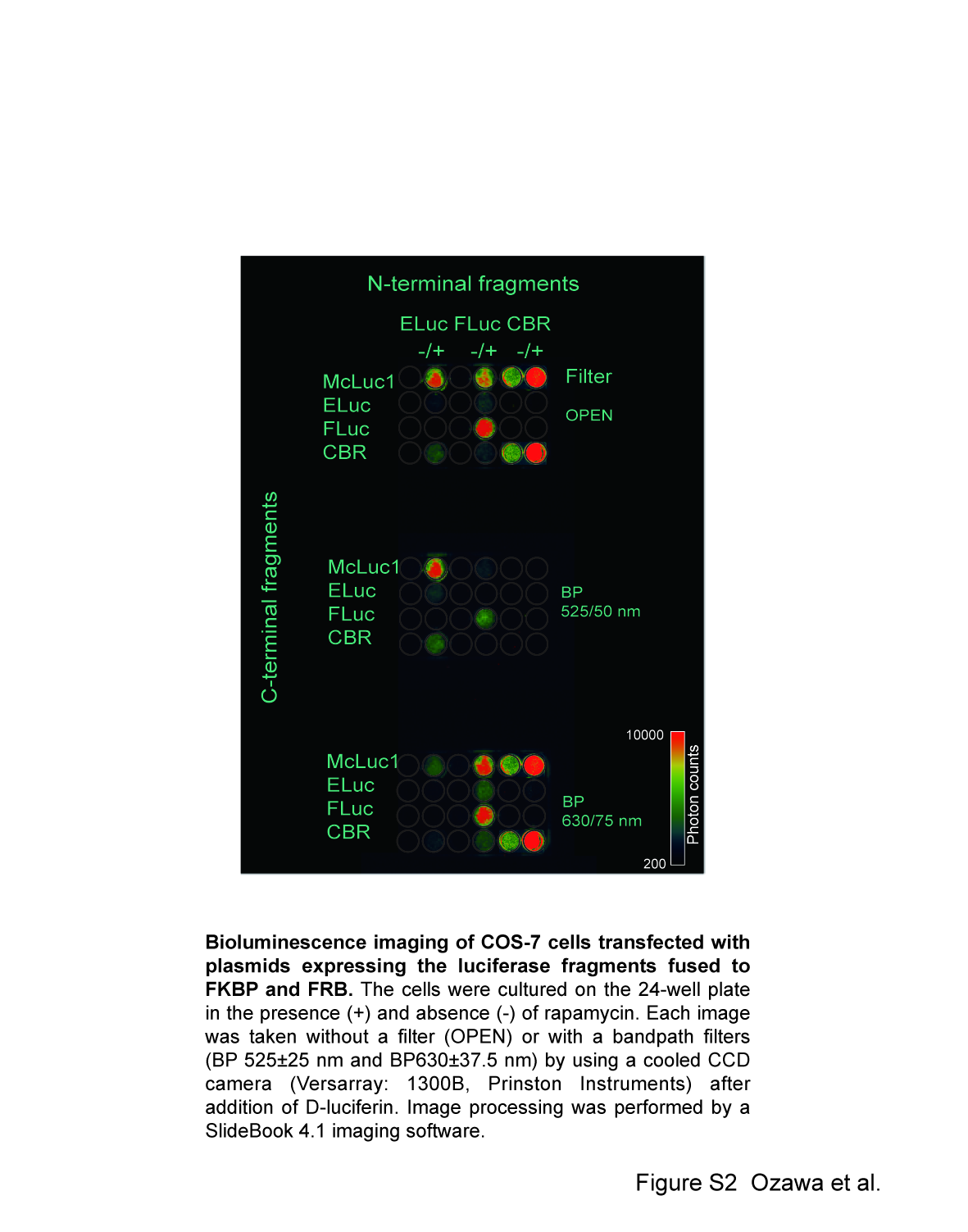

Supplement: Figure S2 — Bioluminescence imaging of COS-7 cells transfected with plasmids expressing the luciferase fragments fused to FKBP and FRB. The cells were cultured on the 24-well plate in the presence (+) and absence (−) of rapamycin. Each image was taken without a filter (OPEN) or with a band-path filters (BP 525±25 nm and BP630±37.5 nm) by using a cooled CCD camera (Versarray: 1300B, Prinston Instruments) after addition of D-luciferin. Image processing was performed by a SlideBook 4.1 imaging software. (2.99 MB TIF) [file pone.0005868.s002.tif]

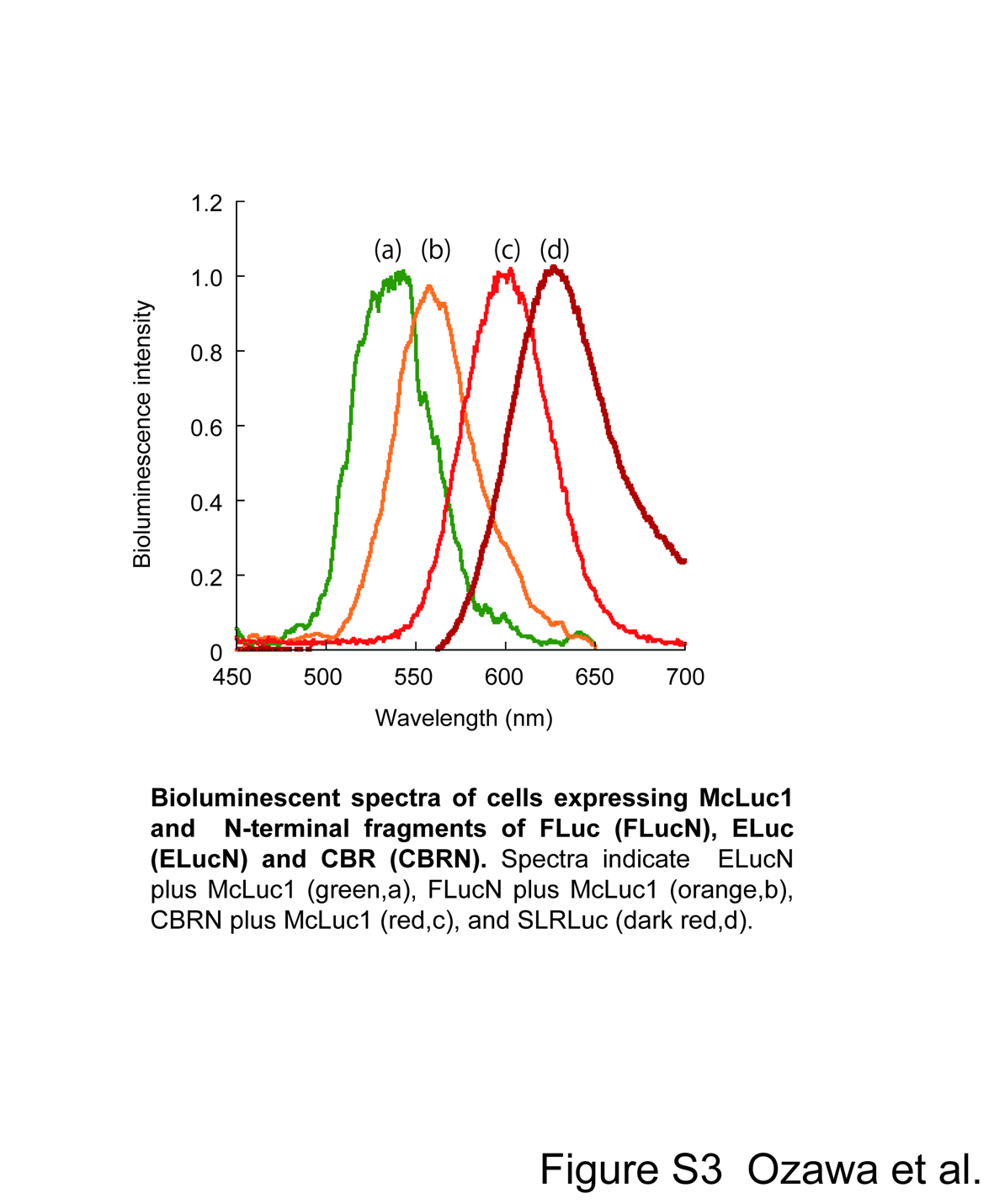

Supplement: Figure S3 — Bioluminescent spectra of cells expressing McLuc1 and N-terminal fragments of FLuc (FLucN), ELuc (ELucN) and CBR (CBRN). Spectra indicate ELucN plus McLuc1 (green, a), FLucN plus McLuc1 (orange, b), CBRN plus McLuc1 (red, c), and SLRLuc (dark red, d). (1.29 MB TIF) [file pone.0005868.s003.tif]

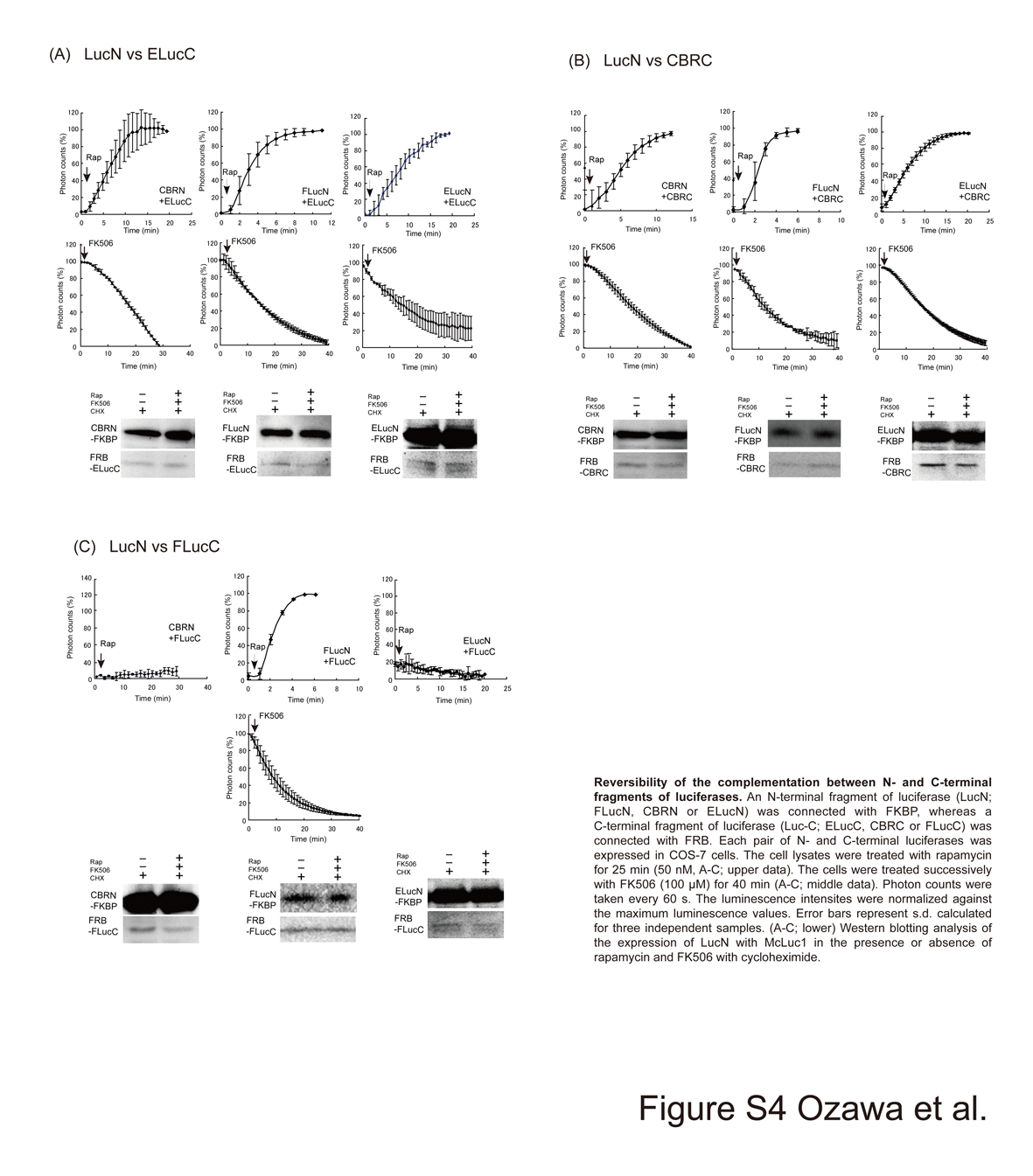

Supplement: Figure S4 — Reversibility of the complementation between N- and C-terminal fragments of luciferases. An N-terminal fragment of luciferase (LucN; FLucN, CBRN or ELucN) was connected with FKBP, whereas a C-terminal fragment of luciferase (Luc-C; ELucC, CBRC or FLucC) was connected with FRB. Each pair of N- and C-terminal luciferases was expressed in COS-7 cells. The cell lysates were treated with rapamycin for 25 min (50 nM, A–C; upper data). The cells were treated successively with FK506 (100 ÂµM) for 40 min (A–C; middle data). Photon counts were taken every 60 s. The luminescence intensites were normalized against the maximum luminescence values. Error bars represent s.d. calculated for three independent samples. (A–C; lower) Western blotting analysis of the expression of LucN with McLuc1 in the presence or absence of rapamycin and FK506 with cycloheximide. (1.30 MB TIF) [file pone.0005868.s004.tif]

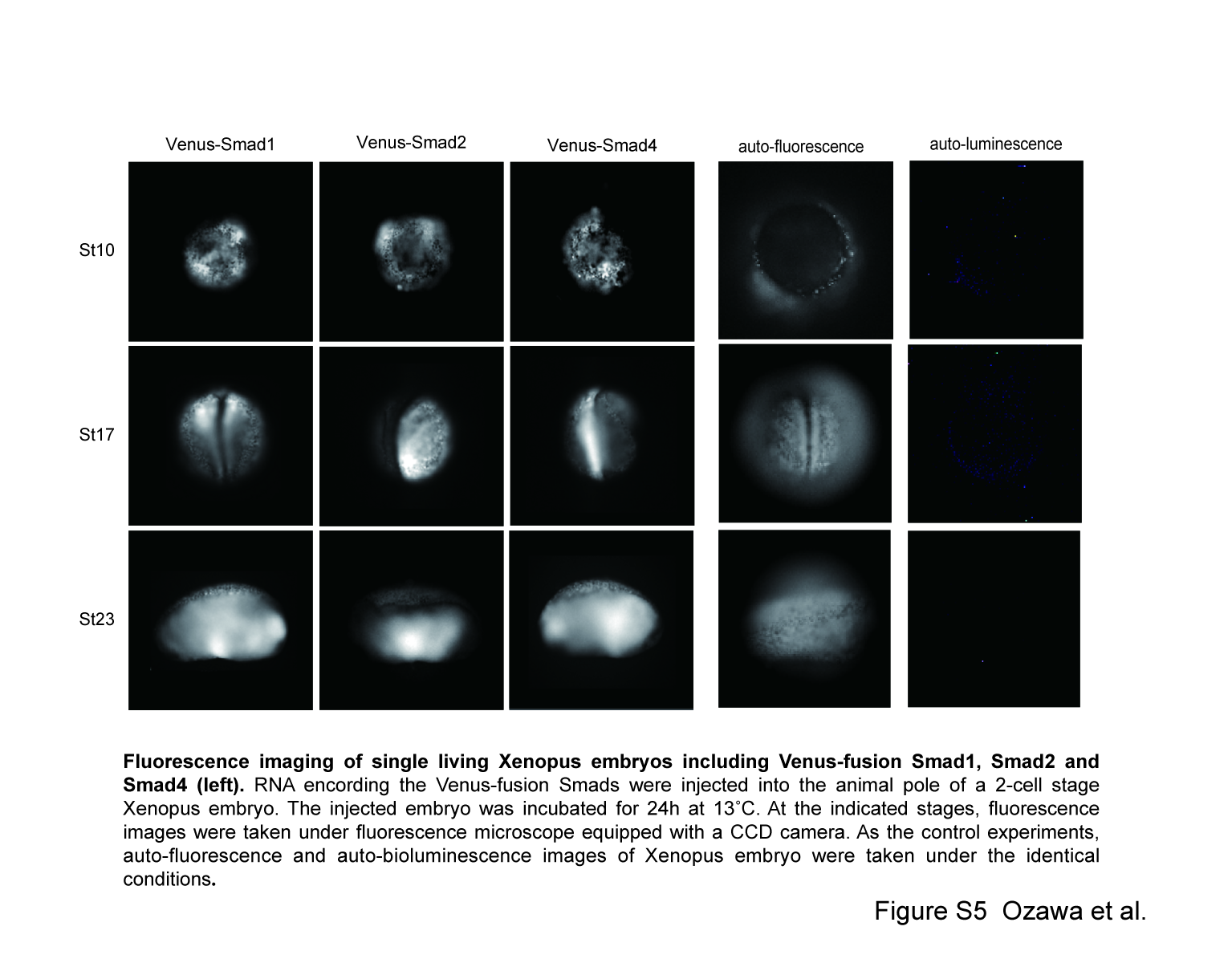

Supplement: Figure S5 — Fluorescence imaging of single living Xenopus embryos including Venus-fusion Smad1, Smad2 and Smad4 (left). RNA encording the Venus-fusion Smads were injected into the animal pole of a 2-cell stage Xenopus embryo. The injected embryo was incubated for 24 h at 13°C. At the indicated stages, fluorescence images were taken under fluorescence microscope equipped with a CCD camera. As the control experiments, auto-fluorescence and auto-bioluminescence images of Xenopus embryo were taken under the identical conditions. (4.94 MB TIF) [file pone.0005868.s005.tif]
