# Supplementary material for: High-Sensitivity Real-Time Imaging of Dual Protein-Protein Interactions in Living Subjects Using Multicolor Luciferases
Source: PLoS One. 2009 Jun 12;4(6):e5868. doi: 10.1371/journal.pone.0005868 (PMC2697115; doi:10.1371/journal.pone.0005868)
Supplement: Table S1 — Comparison of the amino acid sequences between FLuc, CBR and ELuc. (0.20 MB PDF) [file pone.0005868.s006.pdf]

Table S1 Ozawa et al.

Comparison of the amino acid sequences between FLuc, CBR and ELuc.

1st: PpyLuc / 2nd: CBR

1st Amino Acid Sequence
 File Name : PpyLuc
 Sequence Size : 550

2nd Amino Acid Sequence
 File Name : CBR
 Sequence Size : 542

```
[48.8% / 543 aa]
   1'  MEDAKNIKKGPAPFYPLEDGTAGEQLHKAMKRYALVPGTIAFTDAHIEVDITYAEYFEM
        ..**.  **.*..**** **** * .*..... .*   *..*.  . ...* *.**
   1"  MVKREKNVIYGPEPLHPLEDLTAGEMLFRALRKHSHLP--QALVDVVGDESLSYKEFFEA

  60' SVRLAEAMKRYGLNTNHRIVVCSENSLQFFMPVLGALFIGVAVAPANDIYNERELLNSMG
       .* **......* . *. . . .*.**. . **.**..* .**. ***.*. *   ** . **
  59" TVLLAQSLHNCGYKMNDVVSICAENNTRFFIPVIAAWYIGMIVAPVNESYIPDELCKVMG

 120' ISQPTVVFVSKKGLQKILNVQKKLPIIQKIIIMDSKTDYQGFQSMYTFVTSHLPPGFNEY
       **.*.**..*. *.*.*.**..  .*..***.*. .. .* .*. . *. *. . * *
 119" ISKPQIVFTTKNILNKVLEVQSRTNFIKRIIILDTVENIHGCESLPNFI-SRYSDG-NIA

 180' DFVPESFDRDKTIALIMNSSGSTGLPKGVALPHRTACVRFSHARDPIFGNQIIPDTAILS
       .* *  **. . .* *. ***.*******  .*.. ***. ** **  .*.*.**....*
 177" NFKPLHFDPVEQVAAILCSSGTTGLPKGVMQTHQNICVRLIHALDPRYGTQLIPGVTVLV

 240' VVPFHHGFGMFTTLGYLICGFRVVLMYRFEEELFLRSLQDYKIQSALLVPTLFSFFAKST
        .** *.**. .****.. *.**... **..* **...***...*.. **... *..**.
 237" YLPFFHAFGFHITLGYFMVGLRVIMFRRFDQEAFLKAIQDYEVRSVINVPSVILFLSKSP

 300' LIDKYDLSNLHEIASGGAPLSKEVGEAVAKRFHLPGIRQGYGLTETTSAILITPEGDDKP
       *.******.*.*. . *.***.***.*..***..***** *.****.****. *  ... *.
 297" LVDKYDLSSLRELCCGAAPLAKEVAEVAAKRLNLPGIRCGFGLTESTSAIIQTLGDEFKS

 360' GAVGKVVPFFEAKVVDLDTGKTLGVNQRGELCVRGPMIMSGYVNNPEATNALIDKDGWLH
       *..*.*.*...**..* .***.** ** ****..***. .***** ***.. **.*****
 357" GSLGRVTPLMAAKIADRETGKALGPNQVGELCIKGPMVSKGYVNNVEATKEAIDDDGWLH

 420' SGDIAYWDEDEHFFIVDRLKSLIKYKGYQVAPAELESILLQHPNIFDAGVAGLPDDDAGE
       ***..*.******..*** *.****** ********.***..* * *..*.*.** .***
 417" SGDFGYYDEDEHFYVVDRYKELIKYKGSQVAPAELEEILLKNPCIRDVAVVGIPDLEAGE

 480' LPAAVVVLEHGKTMTEKEIVDYVASQVTTAKKLRGGVVFVDEVPKGLTGKLDARK-IREI
       **.* ** ..*...*.**. **.*..*. .* ***** ***..*...***.. .. ....
 477" LPSAFVVKQPGTEITAKEVYDYLAERVSHTKYLRGGVRFVDSIPRNVTGKITRKELLKQL

 539' LIKAKKGGKIAV
       *.**
 537" LVKAGG
```

1st: PpyLuc / 2nd: ELuc

1st Amino Acid Sequence
 File Name : PpyLuc
 Sequence Size : 550

2nd Amino Acid Sequence
 File Name : ELuc
 Sequence Size : 542

```
[47.8% / 540 aa]
   1' MEDAKNIKKGPAPFYPLEDGTAGEQLHKAMKRYALVPGTIAFTDAHIEVDITYAEYFEMS
       **.**.  **.*.**..**** *..*..... .*.*   *.  . ...*.*.*. .
   1" MEREKNVVYGPEPKHPLGNFTAGEMLYNALHKHSHIPQAI--LDVMGNESLSYQEFFDTT

  61' VRLAEAMKRYGLNTNHRIVVCSENSLQFFMPVLGALFIGVAVAPANDIYNERELLNSMGI
       *.*.......* . *. . . .*.**. . **.*...* .**..***.*. *   ** .  **
  59" VKLGQSLQNCGYKMNDVVSICAENNKRFFIPIISAWYIGMVVAPVNEDYIPDELCKVTGI

 121' SQPTVVFVSKKGLQKILNVQKKLPIIQKIIIMDSKTDYQGFQSMYTFVTSHLPPGFNEYD
       *.*..**...* *.*.*.*...   *..***.**...  * .*...*....    ....
 119" SKPILVFTTRKILPKVLEVKDRTNYIKRIIILDSEENLLGCESLHNFMSRYSDNNLQT--

 181' FVPESFDRDKTIALIMNSSGSTGLPKGVALPHRTACVRFSHARDPIFGNQIIPDTAILSV
       * *  .*. . .* *. ***.*******  .**. ***..**.**  *.*.**....*.
 177" FKPLHYDPVDQVAAILCSSGTTGLPKGVMQTHRNICVRLTHASDPRVGTQLIPGVSVLAY

 241' VPFHHGFGMFTTLGYLICGFRVVLMYRFEEELFLRSLQDYKIQSALLVPTLFSFFAKSTL
       .** *.**. ..***.. *.***.. **..*.**...***...*.. **. . *..** *
 237" LPFFHAFGFSINLGYFMVGLRVVMLRRFNQEVFLKAIQDYEVRSVINVPSTILFLSKSPL

 301' IDKYDLSNLHEIASGGAPLSKEVGEAVAKRFHLPGIRQGYGLTETTSAILITPEGDDKPG
       .******.* *. .*.***.***.* ..**..***** ******.***  . * ... *.*
 297" VDKYDLSTLAELCCGAAPLAKEVAEIAVKRLNLPGIRCGYGLTESTSANIHTLHNEFKSG

 361' AVGKVVPFFEAKVVDLDTGKTLGVNQRGELCVRGPMIMSGYVNNPEATNALIDKDGWLHS
       ..***.*...**..* .**..** ** ****..***. .******.**.. **.******
 357" SLGKVTPYMAAKIIDRNTGEALGPNQVGELCIWGPMVTKGYVNNPQATKEAIDDDGWLHS

 421' GDIAYWDEDEHFFIVDRLKSLIKYKGYQVAPAELESILLQHPNIFDAGVAGLPDDDAGEL
       **..*.****.*.**** *.***********.***.******.* *..*.*.** .****
 417" GDFGYYDEDEYFYIVDRYKELIKYKGYQVAPVELEEILLQHPGIRDVAVVGIPDIEAGEL

 481' PAAVVVLEHGKTMTEKEIVDYVASQVTTAKKLRGGVVFVDEVPKGLTGKLDARKIREILI
       **. ** ..*  .*.**. *..*.*. .* ***** ***..*...***.. ...** *.
 477" PAGFVVKQPGAQLTAKEVYDFLAQRVSHSKYLRGGVRFVDSIPRNVTGKISRKELREALM

 541' KAKKGGKIAV

 537" EKASKL
```

1st: ELuc / 2nd: CBR

1st Amino Acid Sequence
 File Name : ELuc
 Sequence Size : 542

2nd Amino Acid Sequence
 File Name : CBR
 Sequence Size : 542

```
[81.3% / 540 aa]
   1'  MEREKNVVYGPEPKHPLGNFTAGEMLYNALHKHSHIPQAILDVMGNESLSYQEFFDTTV
        ..*****.***** ***...******..**.****.***..**.*.*****.***..**
   1" MVKREKNVIYGPEPLHPLEDLTAGEMLFRALRKHSHLPQALVDVVGDESLSYKEFFEATV

  60' KLGQSLQNCGYKMNDVVSICAENNKRFFIPIISAWYIGMVVAPVNEDYIPDELCKVTGIS
        *.***.*****************.*****.*.******.******.********* ***
  61" LLAQSLHNCGYKMNDVVSICAENNTRFFIPVIAAWYIGMIVAPVNESYIPDELCKVMGIS

 120' KPILVFTTRKILPKVLEVKDRTNYIKRIIILDSEENLLGCESLHNFMSRYSDNNLQTFKP
       ** .****..** *****..***.********. **. *****.**.*****.*...***
 121" KPQIVFTTKNILNKVLEVQSRTNFIKRIIILDTVENIHGCESLPNFISRYSDGNIANFKP

 180' LHYDPVDQVAAILCSSGTTGLPKGVMQTHRNICVRLTHASDPRVGTQLIPGVSVLAYLPF
       **.***.*********************.******.** *** ********.**.****
 181" LHFDPVEQVAAILCSSGTTGLPKGVMQTHQNICVRLIHALDPRYGTQLIPGVTVLVYLPF

 240' FHAFGFSINLGYFMVGLRVVMLRRFNQEVFLKAIQDYEVRSVINVPSTILFLSKSPLVDK
       ****** *.**********.*.***.**.******************.************
 241" FHAFGFHITLGYFMVGLRVIMFRRFDQEAFLKAIQDYEVRSVINVPSVILFLSKSPLVDK

 300' YDLSTLAELCCGAAPLAKEVAEIAVKRLNLPGIRCGYGLTESTSANIHTLHNEFKSGSLG
       ****.* ***************.*.***********.******** *.**  .********
 301" YDLSSLRELCCGAAPLAKEVAEVAAKRLNLPGIRCGFGLTESTSAIIQTLGDEFKSGSLG

 360' KVTPYMAAKIIDRNTGEALGPNQVGELCIWGPMVTKGYVNNPQATKEAIDDDGWLHSGDF
       .*** ***** **.**.************ ****.******  .*****************
 361" RVTPLMAAKIADRETGKALGPNQVGELCIKGPMVSKGYVNNVEATKEAIDDDGWLHSGDF

 420' GYYDEDEYFYIVDRYKELIKYKGYQVAPVELEEILLQHPGIRDVAVVGIPDIEAGELPAG
       *******.**.************ ****.*******..* ***********.******..
 421" GYYDEDEHFYVVDRYKELIKYKGSQVAPAELEEILLKNPCIRDVAVVGIPDLEAGELPSA

 480' FVVKQPGAQLTAKEVYDFLAQRVSHSKYLRGGVRFVDSIPRNVTGKISRKELREALMEKA
       *******...*******.**.****.********************.****  ..*. **
 481" FVVKQPGTEITAKEVYDYLAERVSHTKYLRGGVRFVDSIPRNVTGKITRKELLKQLLVKA

 540' SKL
       .
 541" GG
```
