# Supplementary material for: High-Sensitivity Real-Time Imaging of Dual Protein-Protein Interactions in Living Subjects Using Multicolor Luciferases
Source: PLoS One. 2009 Jun 12;4(6):e5868. doi: 10.1371/journal.pone.0005868 (PMC2697115; doi:10.1371/journal.pone.0005868)
Supplement: Table S2 — Maximal bioluminescence intensities of complement luciferase fragments in the presence of rapamycin. (0.01 MB PDF) [file pone.0005868.s007.pdf]

Table S2. Maximal bioluminescence intensities of complement luciferase fragments in the presence of rapamycin.

		N-terminal luciferase fragment		
		FLucN	ELucN	CBRN
C-terminal luciferase fragment	FLucC	$(9.5 \pm 1.9) \times 10^3$	$(2.9 \pm 0.6) \times 10^2$	$(3.1 \pm 0.5) \times 10^2$
	ELucC	$(1.3 \pm 0.3) \times 10^4$	$(3.8 \pm 0.3) \times 10^3$	$(1.8 \pm 0.1) \times 10^5$
	CBRC	$(8.5 \pm 2.4) \times 10^2$	$(1.4 \pm 0.2) \times 10^5$	$(8.5 \pm 0.5) \times 10^5$
	McLuc1	$(1.6 \pm 0.5) \times 10^5$	$(1.7 \pm 2.0) \times 10^5$	$(2.3 \pm 0.2) \times 10^5$
